# Removal of Zinc Ions Using Hydroxyapatite and Study of Ultrasound Behavior of Aqueous Media

**DOI:** 10.3390/ma11081350

**Published:** 2018-08-03

**Authors:** Simona Liliana Iconaru, Mikael Motelica-Heino, Régis Guegan, Mihai Valentin Predoi, Alina Mihaela Prodan, Daniela Predoi

**Affiliations:** 1 National Institute of Materials Physics, Atomistilor Street, No. 405A, P.O. Box MG 07, Magurele 077125, Romania; simonaiconaru@gmail.com; 2 Institut des Sciences de la Terre d’Orléans (ISTO), UMR 7327 CNRS Université d’Orléans, 1A rue de la Férollerie, 45071 Orléans CEDEX 2, France; mikael.motelica@univ-orleans.fr; 3 Faculty of Science and Engineering, Global Center for Science and Engineering, Waseda University, 3-4-1, Okubo, Shinjuku-ku, Tokyo 169-8555, Japan; regis.guegan@aoni.waseda.jp; 4 Department of Mechanics, University Politehnica of Bucharest, BN 002, 313 Splaiul Independentei, Sector 6, Bucharest 060042, Romania; predoi@gmail.com; 5 Emergency Hospital Floreasca Bucharest, 8 Calea Floreasca, Sector 1, Bucharest 014461, Romania; prodan1084@gmail.com; 6 Carol Davila University of Medicine and Pharmacy, 8 Eroii Sanitari, Sector 5, Bucharest 050474, Romania

**Keywords:** hydroxyapatite, ultrasonic measurements, water depollution, zinc, adsorption

## Abstract

The present study demonstrates the effectiveness of hydroxyapatite nanopowders in the adsorption of zinc in aqueous solutions. The synthesized hydroxyapatites before (HAp) and after the adsorption of zinc (at a concentration of 50 mg/L) in solution (HApD) were characterized using X-ray diffraction (XRD), and scanning and transmission electron microscopy (SEM and TEM, respectively). The effectiveness of hydroxyapatite nanopowders in the adsorption of zinc in aqueous solutions was stressed out through ultrasonic measurements. Both Langmuir and Freundlich models properly fitted on a wide range of concentration the equilibrium adsorption isotherms, allowing us to precisely quantify the affinity of zinc to hydroxyapatite nanopowders and to probe the efficacy of hydroxyapatite in removal of zinc ions from aqueous solutions in ultrasonic conditions.

## 1. Introduction

The most pressing issue today is the increase in contaminated sites in both numbers and area as a result of rapidly growing industrial activities that have led to ecosystem damage due to excessive pollution. Moreover, many industries have the potential to pollute water resources with different wastes such as organic and inorganic compounds. The most frequent pollutants due to the industrial sites are heavy metal ions and aromatic compounds [1,2]. Due to an increased awareness regarding the well-being of the planet, tremendous attention has been paid to heavy metals such as cadmium, lead, zinc, and arsenic, which are reported to have a detrimental impact on the environment all over the world. These particular heavy metals are not biodegradable and can be accumulated in organisms, thus representing a serious health threat to plants, animals and human beings. Indeed, metals, such as zinc, cadmium, led, and arsenic, show a high toxicity even at low concentrations. Even though human do not have direct contact with these pollutants, human health can be indirectly influenced by multiple ways, particularly through drinking water and the food chain. In addition, in agriculture, the presence of heavy metals pollution in soils can cause great harm to crop growth, yield and quality. Therefore, the removal of heavy metals, from natural waters or soils, has attracted considerable attention [3,4]. Various conventional technologies for the removal of heavy metal ions from aqueous solution, such as chemical precipitation, electrochemical treatment, ion exchange, reverse osmosis and electrodialysis, have been described [5]. Recently, considerable attention has been given to alternative methods, one of the most promising being the use of cheap materials as potential sorbents for heavy metals removal [6,7,8]. The most popular materials used as sorbents have been carbons, zeolites, clays, biomass and polymeric materials [9]. However, in the literature, it has been reported that these materials exhibit low adsorption capacities towards heavy metals ions and suffers from separation inconvenience. Therefore, considerable efforts are still needed for the development of newly materials that can be used as adsorbents in decontamination applications. Several studies conducted in recent years have reported the applications of mineral materials in the disposal of wastewaters contaminated with heavy metals [9].

Materials such as zeolite [10], clay minerals [11,12] and other organo-derived clay materials [13,14,15] have been reported to be promising candidates in the treatment of wastewater due to their excellent surface characteristics. Recent studies have reported that phosphate minerals represent promising materials in the treatment of wastewaters for the removal of fluoride and heavy metals [16,17]. Incentive results on the efficacy of phosphate minerals have been reported for the activity of hydroxyapatite (HAp) with the chemical formula Ca_10_(PO_4_)_6_(OH)_2_ as an excellent material for removing long-term pollutants from contaminated water due to its high affinity for heavy metals, low water solubility, high stability and low cost [18].

One of the most popular and widely used adsorbents in water treatment especially for heavy metal removal has been activated carbon since it was introduced [19]. Even though activated carbon [20], silica gel [21] and activated alumina [22] are highly effective for the adsorption of heavy metals from water, their use is restricted, as these materials remain expensive materials. Therefore, they cannot be widely used in small-scale industries because of cost inefficiency. Recently, the interest in the research of alternative adsorbents to replace the costly activated carbon, silica gel and activated alumina has increased. Considerable attention has been focused on adsorbents, with metal-binding capacities and able to remove heavy metals from contaminated water at low cost [23,24]. Natural materials such as chitosan, zeolites, chitosan, clay, or waste products from industrial operations such as fly ash, coal, and oxides have been classified as low-cost adsorbents [25]. It was estimated that chitosan could be obtained using fish and crustaceans as raw materials at a market price of US$ 15.43/kg [26]. Similar estimations have been made regarding the price of zeolites which are considered very cheap (about US$ 0.03–0.12/kg) [27]. On the other hand, apatite materials have been depicted as ideal materials for long-term containment of pollutants due to their high sorption capacity for actinides and heavy metals [28], low water solubility [29], high stability under reducing and oxidizing conditions, availability and low cost [30]. Moreover, natural hydroxyapatite obtained from bovine cortical bone ash [30] has been considered as an environmentally benign functional material and has been widely investigated for water treatment applications due to its high capacity for the removal of heavy metal ions, low water solubility, high stability under reducing and oxidizing conditions, availability and low cost [31,32,33].

Zinc is considered one of the most important elements and plays an essential role in biological functions. Nevertheless, zinc ions at a certain concentration display a great toxicity towards human organism [34]. Thus, the Canadian Water Quality Guidelines [35] and WHO [36] defined that the maximum admissible level of zinc in drinking water be ≤5 mg/L. The contamination of zinc in effluents often results from industry discharges. Since zinc is not biodegradable, it travels through food chain via bioaccumulation and possess a high toxicity risk to humans [37]. In the last years, the use of ultrasound measurements to characterize suspensions has become a large and growing application, as reviewed by McClements [38]. Several studies have stated that ultrasonic measurements could offer significant insight about materials properties [39,40]. Even though there have been reported only a handful of few basic and quantitative investigations, the common conclusion of the reported papers has been that ultrasound methods are very rigorous and much less sensitive to contamination providing accurate information about the microstructural properties as well as deformation processes of the investigated samples [41,42].

In this context, this study focused on using hydroxyapatite nanopowders for the removal of zinc ions from contaminated waters. It principally aimed at pointing out the interests of hydroxyapatites as potential adsorbents for zinc (Zn^2+^) in aqueous solution. Pure hydroxyapatite obtained by an adapted coprecipitated method was characterized by XRD, SEM and TEM before and after removing zinc from aqueous solutions. Adsorption kinetics of zinc ions (Zn^2+^) on hydroxyapatite was studied by the Langmuir and Freundlich models. The effectiveness of decontamination was correlated with ultrasonic measurements. The advantages of ultrasonic techniques are synthetically presented in the book of Povey [43] and an updated review was presented by Povey [44]. More recently, Galaz [45] presented an experimental verification of ultrasonic methods applied to suspensions of small particles. There are even ultrasonic studies dedicated to the estimation of the suspension particle sizes, from which we mention here on of the most recent [46]. In this paper, the ultrasonic technique is simple and directly applicable, based on acoustic signature of the signal in different suspensions. The only limitation is that the reference fluid and the depolluted suspension must be tested with the same experimental setup.

## 2. Experimental Section

### 2.1. Synthesis of Hydroxyapatite Nanoparticles (HAp)

The hydroxyapatite, Ca_10_(PO_4_)_6_(OH)_2_, nanopowders were obtained using a previously described adapted coprecipitation method [47,48]. The molar ratio Ca:P was maintained at 1.67 [36].

### 2.2. Structural and Morphological Characterizations

The structure and morphology of the recovered powders were characterized by XRD, SEM and TEM.

X-ray diffraction was performed for HAp nanopowders before and after zinc ions adsorption from aqueous solution using a Bruker D8 Advance diffractometer (Bruker, Karlsruhe, Germany) with nickel filtered CuK𝛼 (λ = 1.5418 Å) radiation and a high efficiency one-dimensional detector (Lynx Eye type, Bruker, Karlsruhe, Germany) operated in integration mode. The data were collected over the 2θ range of 25–55°, using a step size of 0.02° and 34 s measuring time per step.

The morphology of the HAp nanopowders before and after zinc ions adsorption from aqueous solution was investigated by scanning electron microscopy (SEM) using a HITACHI S4500 microscope (Hitachi High Technologies America, Inc., Schaumburg, IL, USA).

The size and morphology of HAp nanopowders before and after zinc ions adsorption from aqueous solution were analyzed by transmission electron microscopy (TEM) using a CM 20 (Philips—FEI, Eindhoven, The Netherlands), equipped with a filament Lab6 that works at 200 kV. For TEM investigations, the nanopowders were dispersed in ethanol using an ultrasonic bath (Retsch GmbH, Haan, Germany) and a drop of the resulting solution was deposited on a carbon-coated Cu grid.

### 2.3. Batch Adsorption Experiments

The effectiveness of zinc adsorption onto hydroxyapatite nanopowders was investigated by batch adsorption experiments. The experiments were conducted in 40 mL silicon tubes with aqueous solutions containing zinc ions in a concentration range of 5–150 mg L^−1^. The amount of the HAp nanopowder used as adsorbent was 0.2 g and the solution pH was adjusted to 5 with a 0.1 M hydrochloric acid (HCl) solution. During the experiments, the solution volume was kept at 20 mL and the mixture was stirred using a Mixer SRT1 Roller (Stuart Scientific, Staffordshire, UK) for 24 h. After 24 h, the tubes were centrifuged for 1 h at 10,000 rpm. The supernatant was filtered, recovered and analyzed by Atomic Absorption Spectrometry (AAS) [49]. The batch experiments were carried out at room temperature, in triplicate. The AAS measurements were performed using a 213.9 nm wavelength for zinc.

### 2.4. Ultrasonic Measurements

**Figure 1:**
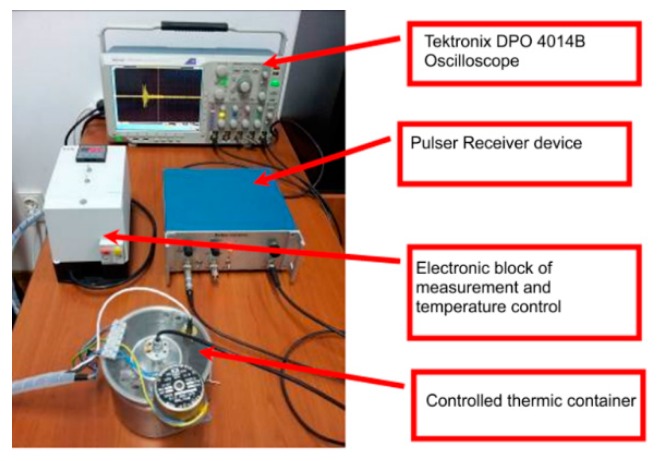
Overview of the chain of measurement.

Ultrasonic measurements were performed on water (Wi), water contaminated with zinc (50 mg/L zinc ions) (WZn) and water after decontamination (Wd). Solutions analyzed by ultrasonic measurements, water (Wi), zinc contaminated water (50 mg/L zinc ions) (WZn) and water after decontamination (Wd) were placed in the thermally controlled container. The temperature of the solutions was kept constant at 21 °C. During the measurements, the WZn and Wd samples were stable and no precipitation of nanoparticles was observed at the end of the experiment. The chain of measurement used to ultrasonic characterization of Wi, WZn, and Wd is shown in Figure 1.

## 3. Results and Discussions

**Figure 2:**
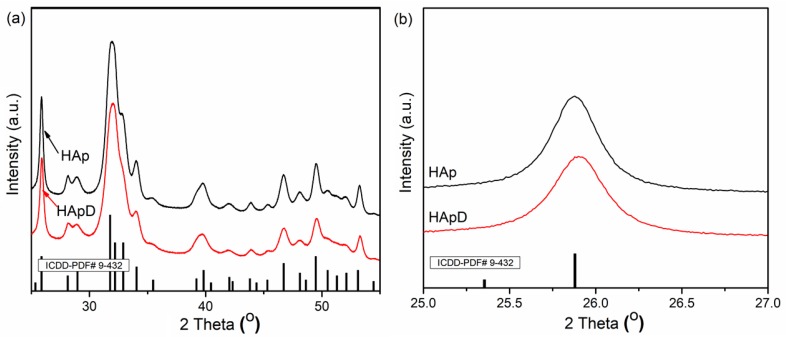
XRD patterns of hydroxyapatite (HAp) and hydroxyapatite recovered after zinc adsorption from aqueous solutions (HApD) containing concentration of 50 mg/L zinc ions. (**a**) 2θ range of 25–55°; (**b**) 2θ range of 25–27°.

To highlight the effectiveness of hydroxyapatite in the removal of zinc ions from aqueous solutions, XRD, SEM, EDX and TEM analysis were performed on prepared hydroxyapatite (HAp) and hydroxyapatite recovered after adsorption of zinc ions from aqueous solutions (HApD). Figure 2a shows the XRD patterns of the HAp and HApD after zinc adsorption from aqueous solutions containing zinc ions in a concentration of 50 mg/L. The XRD peaks from X-ray diffraction patterns of prepared HAp and HApD corresponds to the standard XRD peaks of hexagonal hydroxyapatite (JCPDS 9-432). XRD analysis did not reveal other calcium phosphate phases or mineral formation in the solid of Zn reaction with phosphate in agreement with previous studies conducted by Cao et al. [50]. The crystal size was determined based on Scherer’s formula (D = 0.9λ/βcosθ). In Scherer’s formula, λ is the wave length of X-rays, β is full width at half height of peak in radians and θ is the angle of diffraction. To estimate the mean crystallite size (D) of the HAp and HApD samples, the line broadening of the peaks at 2θ = 25.8° assigned to the crystalline plane (002) was used. The crystal size of HAp determined using the Scherer’s formula was 18.8 ± 1 nm. The D value of the HApD was 15.12 ± 1 nm. The characteristic diffraction peaks such as (002), (210), (211), (300), (202), (310), (222), (213), and (004) can be clearly seen, indicating the formation of HAp crystalline phase. The XRD patterns of HApD showed a similar curve. We observed that the peak associated to (002) diffraction of the HApD shifted toward the high diffraction angle (Figure 2b). According to Hayakawa et al. [51], this behavior suggested that the Zn^2+^ ions were substituted for the Ca^2+^ ion.

**Figure 3:**
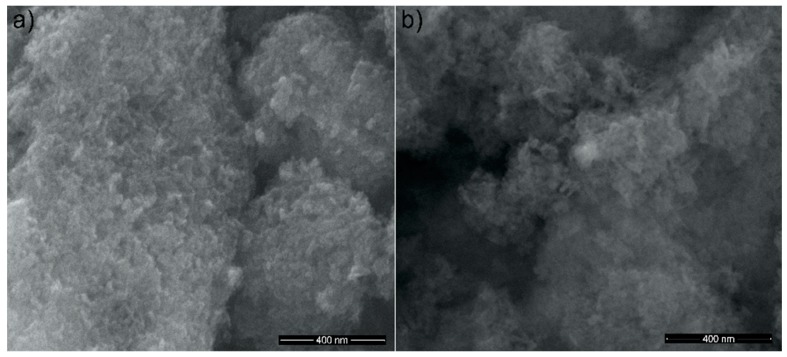
SEM images of HAp (**a**); and hydroxyapatite recovered after zinc adsorption from aqueous solutions (HApD) containing concentration of 50 mg/L zinc ions (**b**).

SEM images of the HAp powders before and after zinc adsorption from aqueous solutions containing zinc ions in a concentration of 50 mg/L are presented in Figure 3a,b. The SEM images revealed that all investigated samples presented nanoparticles with elongated morphology and a tendency of forming agglomerates due to their nanometric size. Furthermore, SEM images suggested that the morphology of the samples was slightly influenced by the presence of the zinc ions in the powders.

**Figure 4:**
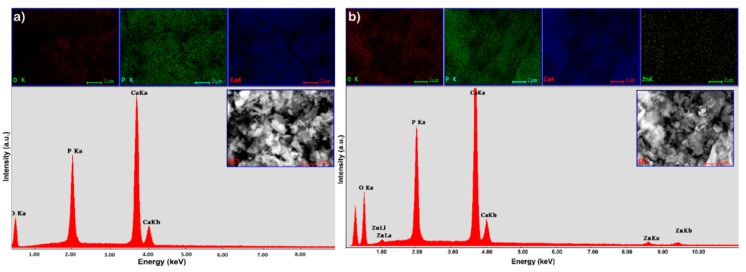
EDX spectrum and elemental mapping of synthetized HAp (**a**); and EDX spectrum and elemental mapping of hydroxyapatite recovered after zinc adsorption from aqueous solutions (HApD) containing concentration of 50 mg/L zinc ions (**b**).

In the EDX spectra of Hap, the presence of peaks for Ca, P and O, the major constituent elements of hydroxyapatite, have been highlighted (Figure 4a). In addition to HAp’s constituents, the EDX spectrum of HApD reveals the presence of zinc (Figure 4b). By the presence of the maxima attributed to zinc in the EDX spectrum of HApD, we can conclude that zinc ions were efficiently adsorbed by synthetized HAp.

**Figure 5:**
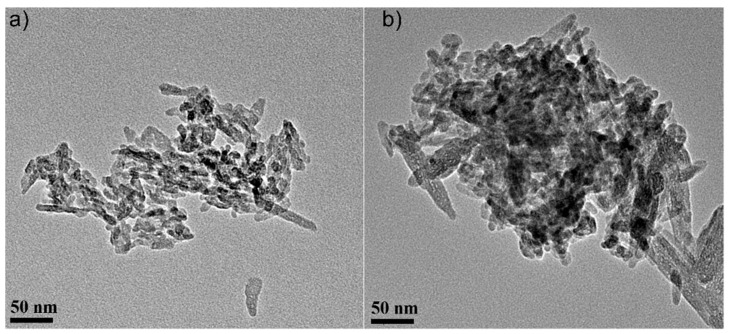
Transmission electron microscopy (TEM) micrographs of the HAp powders before (**a**) and after zinc adsorption from aqueous solutions containing zinc ions (HApD) in a concentration of 50 mg/L (**b**). Scale bar: 50 nm.

In addition, the elemental mapping of HAp and HApD, as shown in Figure 4 and Figure 5, provided information about the homogeneity of the samples and the uniform distribution of the Ca, P and O. The uniform distribution of Zn was also observed in HApD. These results were fully consistent with X-ray diffraction analysis.

Figure 5 presents the transmission electron micrographs of pure hydroxyapatite (HAp) and hydroxyapatite recovered after zinc adsorption from aqueous solutions (HApD) containing zinc ions in a concentration of 50 mg/L. The average particle size of the pure HAp was 20 ± 1 nm. The average particle size of the HApD decreased to 17 ± 1 nm, in agreement with the X-ray results. The particles are less elongated. Furthermore, it should be noted that more agglomeration of the particles after Zn ion adsorption was observed. The particle size and morphology changed little after zinc adsorption from aqueous solutions containing 50 mg/L zinc ions.

The adsorption data obtained from the batch equilibrium experiments were used in the kinetic studies and the applicability of different adsorption isotherms to Zn^2+^ were studied. The percentage removal efficiency of the adsorbents R(%) and the sorption capacity at equilibrium were calculated using the following Equations (1) and (2):

(1) $$\;R\left(\%\right)=\frac{C_{0}-C_{e}}{C_{0}}\times 100\;$$

(2) $$\;q_{e}=\left(C_{0}-C_{e}\right)\times \frac{V}{m}\;$$

where C_0_ (mg/L) is the initial metal ion concentration, C_e_ (mg/L) is the equilibrium concentration of Zn (II), V (L) is the volume of the solution and m (g) is the mass of the adsorbent.

**Figure 6:**
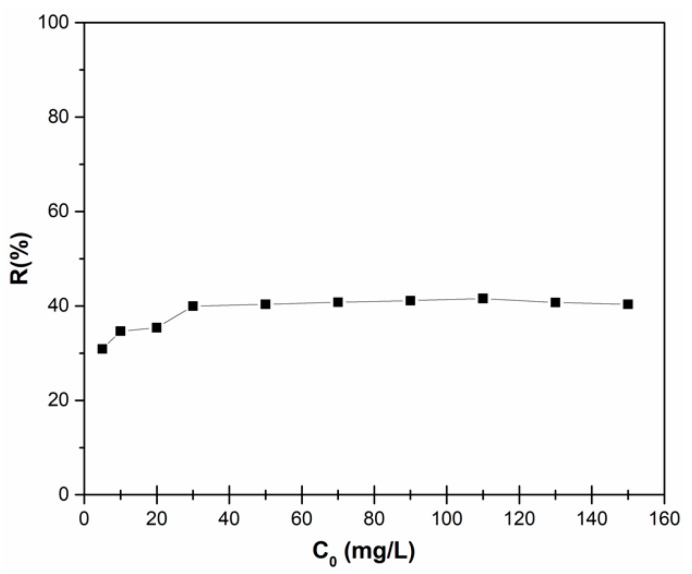
Removal efficiency of zinc ions on HAp depending on the initial zinc concentration.

The adsorption of zinc ions from aqueous solutions using HAp nanopowders was studied in batch experiments and the removal efficiency of adsorption of zinc ions from aqueous solution by HAp is presented in Figure 6. The initial zinc ions concentration was set in the range of 5–150 mg/L. In Figure 6, the adsorption capacity increases with the increase of the initial zinc ions concentration. By increasing the initial concentration of zinc, the interaction between adsorbent and zinc ions is enhanced, leading to an increase in the adsorption uptake of zinc ions onto HAp nanopowders. Figure 6 shows that the percentage zinc ions removal increased from 30.88% to 41.57% with the increase of the initial zinc concentration from 5 mg/L to 150 mg/L. It was also noticed that, after the concentration of 110 mg/L, the removal percentage stabilized around the value of 40%.

The values corresponding to the percentage of zinc ions removal and the adsorption capacity as a function of zinc concentrations at equilibrium are presented in Table 1. The results show that the adsorption capacity of HAp nanopowders increases with the increase of the zinc initial concentration.

The equilibrium interactions between adsorbent and adsorbate are usually described with the aid of sorption isotherms [52]. The experimental data obtained from the batch equilibrium experiments were fitted using Langmuir and Freundlich isotherm equations. Langmuir isotherm is generally constructed with the assumption that a fixed number of adsorption sites are available and that the adsorption is reversible; therefore, this model can be used only when the adsorbent surface is homogeneous. The Langmuir isotherm is expressed as described by Langmuir [53]:

(3) $$\;\frac{C_{e}}{q_{e}}=\frac{C_{e}}{q_{m}}+\frac{1}{K_{L}\times q_{m}}\;$$

where C_e_ is the equilibrium concentration of heavy metal ions (mg/L), q_e_ is the equilibrium adsorption uptake of heavy metal ions (mg/g), q_m_ is the amount adsorbed to form a complete monolayer on the surface (mg/g) and K_L_ is the Langmuir constant (L/mg).

Meanwhile, the Freundlich model is based on the assumption that the adsorption takes place on a heterogeneous surface. The Freundlich isotherm is describe by the following equation [54]:

(4)  lnqe=lnKF+1nlnCe 

where q_e_ is the amount that was adsorbed at the equilibrium concentration (mg/g), K_F_ is an empirical constant of Freundlich isotherm (L/mg), C_e_ is the equilibrium concentration of zinc ions in solution (mg/L) and n is a parameter correlated with the intensity of adsorption.

**Figure 7:**
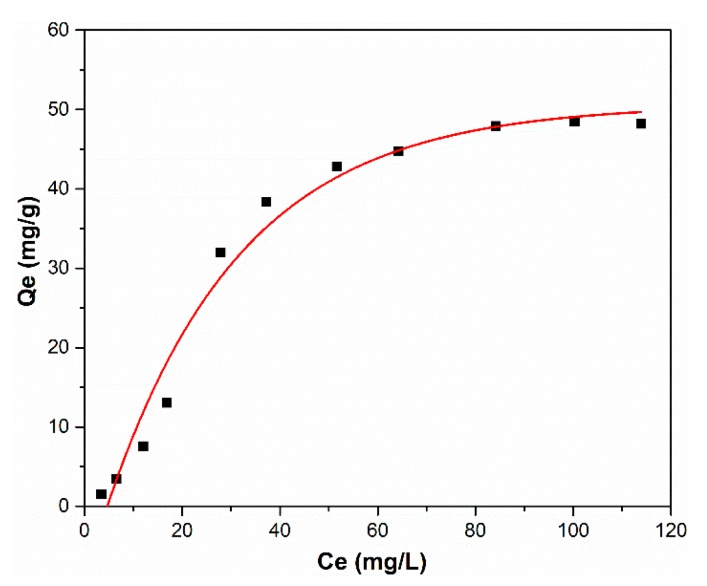
Equilibrium adsorption isotherm for zinc ions onto HAp at room temperature.

For a better understanding of the adsorption capacity of HAp nanopowders, the equilibrium adsorption isotherm for zinc ions onto HAp at room temperature is presented in Figure 7. Figure 7 shows the adsorption capacity of zinc ions upon varying the zinc ions concentration in the aqueous solutions.

**Figure 8:**
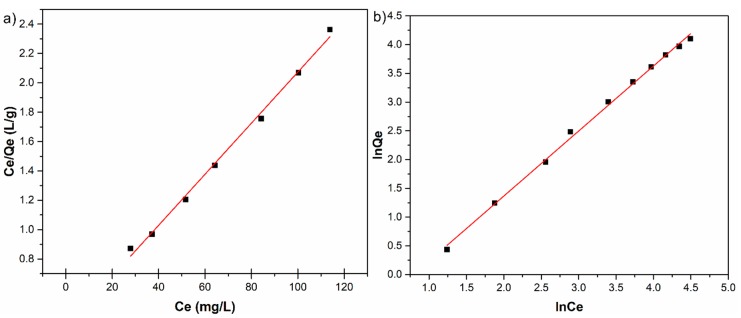
Langmuir (**a**); and Freundlich (**b**) linearized fits for the adsorption of zinc ions on HAp.

The Langmuir and Freundlich linearized fits for the adsorption of zinc ions on HAp are shown in Figure 8. The constants calculated from the linearized plots are reported in Table 2.

At room temperature, the correlation coefficient of Freundlich isotherm for Zn^2+^ removal by HAp was 0.997 and had a higher value than the correlation coefficient of Langmuir isotherm, which was 0.995. On the other hand, the maximum adsorption capacity for the solid phase calculated from the linearized form of Langmuir equation, q_m_, for Zn^2+^ was 57.504 mg/g and the Langmuir constant K_L_ for the adsorption of Zn^2+^ was 0.052 L/mg. The linear plot of C_e_/Q_e_ against C_e_ for the adsorption of zinc ions on HAp exhibited in Figure 8a revealed that the adsorption is in good agreement with the Langmuir model.

By fitting the data using the Freundlich adsorption isotherm model, a linear relationship was obtained where K_F_ has been equal to 0.408, and the 1/n was equal to 1.13 (Figure 8b). Following these results, the inverse of the empirical parameter related to the intensity of adsorption has values greater than 1 (1/n > 1) which shows that the absorption coefficient increases with increase in concentration resulted as effect of increase in hydrophobic surface characteristics after monolayer. The results suggest that HAp nanopowders exhibited a good affinity towards zinc ions, rendering HAp a suitable promising adsorbent for water treatment technologies.

For the first time, the effectiveness of hydroxyapatite in water decontamination was highlighted by ultrasonic studies on water (Wi), zinc-contaminated water (50 mg/L zinc ions) (WZn) and water after decontamination (Wd). The parameters confirming the decontamination are the signal velocity in the suspension and the similarity of the acoustic signals of decontaminated water and pure water. For the interpretation of the ultrasonic signals, specialized programs have been used, able to provide essential information on the parameters characteristic of each studied material. For the ultrasonic characterization of the dispersions presented above (Wi, HZ2 and Wf) it was considered that the propagation velocity of the acoustic waves in homogeneous liquids can be calculated with the relation:

(5) $$c=\sqrt{\frac{1}{\kappa \rho }}\;$$

where the adiabatic compressibility κ is the inverse of the compression elastic modulus (K=1/κ). The ultrasound velocity through solutions and suspensions, in the linear approximation of small disturbances, depends on the average density and average compressibility. These averaged values are calculated from the Urick equations [55]:

(6) $$\;\kappa =\sum_{i}\Phi_{i}\kappa_{i};\;\rho =\sum_{i}\Phi_{i}\rho_{i}\;$$

in which Φ*_i_* represents the volumetric fraction of component of index *i*. The volumetric fraction can be transformed into mass fraction by:

(7) $$w=\frac{\Phi \rho_{2}}{\Phi \rho_{2}+\left(1-\Phi \right)\rho_{1}}\;$$

in thecase of two phases, marked by index 1 for the solvent and 2 for the dispersed phase [43,44]:

(8) $$\kappa =\Phi \kappa_{2}+\left(1-\Phi \right)\kappa_{1};\;\rho =\Phi \rho_{2}+\left(1-\Phi \right)\rho_{1}\;$$

It is known that water has a different ultrasonic acoustic behavior in many aspects from other fluids. For example, below 74 °C, the gradient of ultrasound propagation velocity in water is positive, while for most liquids it is negative. Another special property is the negative dilatation coefficient below 4 °C. However, many experimental results are available for water, which facilitates studies based on this solvent. Moreover, the ultrasound speed in water strongly depends on the temperature.

**Figure 9:**
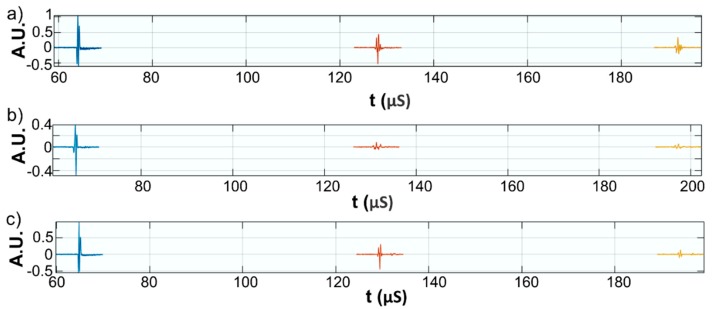
Acquired signals for: Wi (**a**); WZn (**b**); and Wd (**c**).

**Figure 10:**
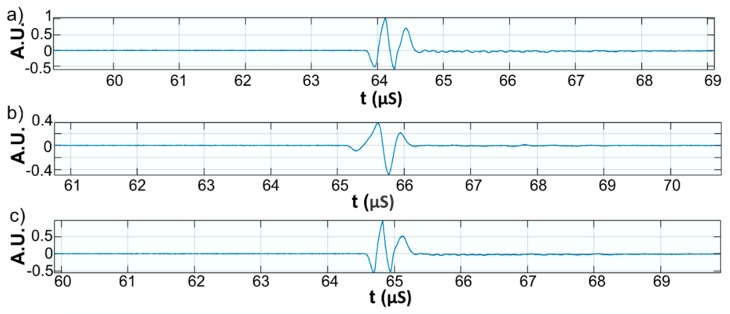
Zoomed first echo for: Wi (**a**); WZn (**b**); and Wd (**c**).

**Figure 11:**
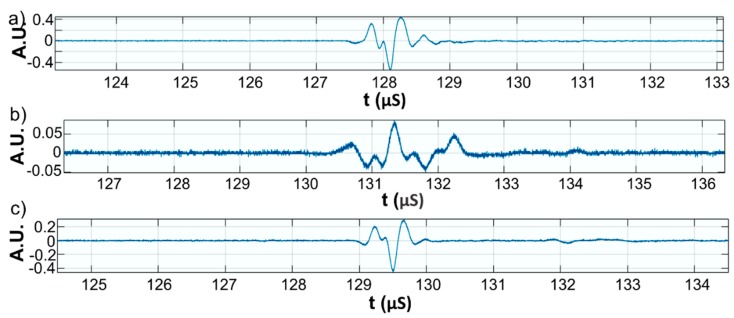
Zoomed second echo for: Wi (**a**); WZn (**b**); and Wd (**c**).

**Figure 12:**
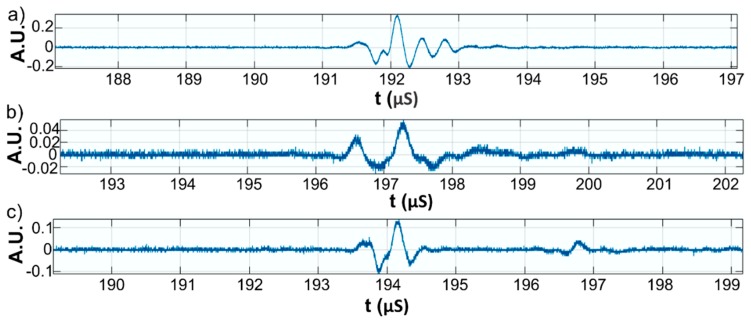
Zoomed third echo for: Wi (**a**); WZn (**b**); and Wd (**c**).

This paper presents the experimental results of ultrasonic measurements on water (Wi), zinc contaminated water with 50 mg/L zinc ions (WZn) and water after decontamination (Wd). The time intervals between echoes make it possible to accurately determine the distance between the transducer face and the flat bottom surface of the used aluminum container. The obtained signals were processed using a specialized algorithm implemented in Matlab (Matlab2016). The modeling of the ultrasound wave effect on the analyzed samples was performed with an original model implemented in Comsol–Multiphysics finite elements program. The specialized program for temporal signal analysis allows determining the time differences between equivalent echoes in different fluids, with an accuracy of 1 ns. For all tested fluids, the reference was bi-distilled water. The small signals observed before the second and third echoes are signals of multiple reflections on the flat bottom of the aluminum container used for the fluids tested. These signals do not affect the results obtained in the signal interpretation program. Figure 9 shows the signals acquired for Wi, WZn and Wd. Figure 10, Figure 11 and Figure 12 present the first, second and third echoes for the three studied samples, respectively.

The time differences between each of the three echoes obtained for Wi e and WZn were obtained. Thus, in the case of the first echo recorded for Wi and WZn, there was a temporal difference of 1.67 μs. For the second echo, the temporal difference was 3.255 μs, which corresponds to a difference in equivalent signals of 1.628 μs. Similarly, for the third echo, the time difference to this echo was 5.17 μs, thus obtaining a temporal difference of 1.723 μs. Using the mean of these time differences and the distance traveled by the signals, the ultrasonic velocity was obtained by WZn (1454.17 ± 1.06 m/s) and an attenuation of 9.11055 Np/m (1.04889 dB/m). In addition, the time differences between each of the three echoes obtained for Wi and Wd were obtained. Comparing the first recorded echo for bi-distilled water and Wd dispersion, a time difference of 0.703 μs was obtained. For the second echo, the time difference was 0.701 μs. Similarly, for the third echo, the temporal difference was 0.691 μs. Considering these temporal differences, ultrasonic velocity was obtained as 1476.02 ± 0.15m/s for Wd and a corresponding attenuation of 3.87419 Np/m (0.446033 dB/m).

**Figure 13:**
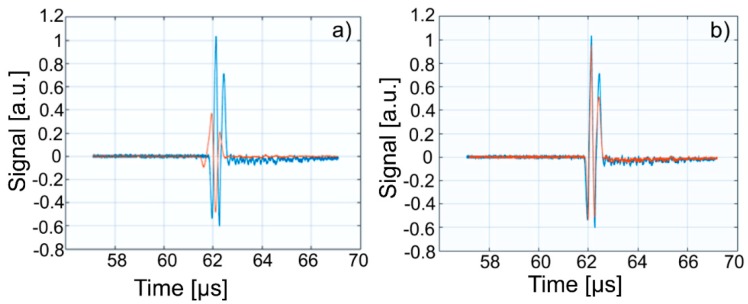
Overlapping of the first echo of Wi (blue) over signals from: WZn (**a**); and Wd (**b**) (orange).

**Figure 14:**
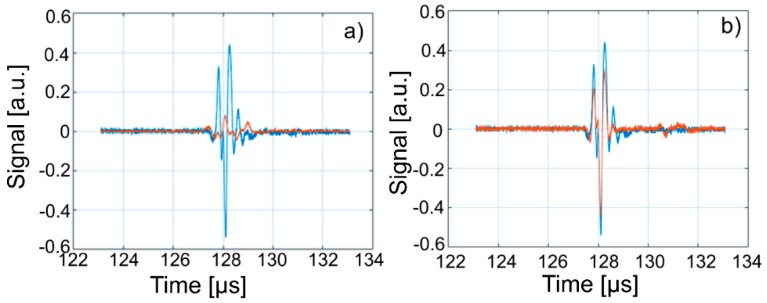
Overlapping of the second echo of Wi (blue) over signals from: WZn (**a**); and Wd (**b**) (orange).

**Figure 15:**
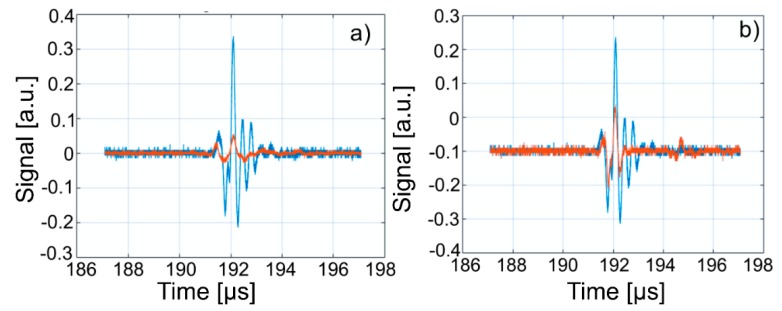
Overlapping of third echo of Wi (blue) over signals from: WZn (**a**); and Wd (**b**) (orange).

Figure 13, Figure 14 and Figure 15 show overlapping of the three echoes obtained for Wi and WZn. The smaller signals, due to multiple reflections on the flat bottom of the aluminum container superposed over those of Wi (blue signals), can be seen. The almost disappearance of these low intensity signals in the case of WZn dispersions (orange signals) is determined by the increased ultrasonic signal attenuation for this medium. In overlapping these signals, the time delay is optimized for overlapping the signal modules, avoiding problems of phase jumps of the signal at the successive reflections on the aluminum bottom wall.

The intensities of WZn signals are much weaker than those associated with Wi. This is due to the increased attenuation through the investigated dispersions. In the case of decontamination water (Wd), the three signals resemble those of the Wi-associated signals, even if they are slightly attenuated. This behavior reveals that the amount of zinc in the Wd sample dropped significantly, since the decontaminated water has ultrasonic characteristics (velocity, signal aspect) closely resembling those of pure water. A great advantage of ultrasound measurements that were first used in such a study is that this method is reproducible and non-destructive. This method offers a quick analysis with low costs, as the consumption of resources such as water, electricity, etc. is low. On the other hand, the analyzed material (liquid or solid) is not damaged during the analysis and can be examined in the operative condition. The results obtained by ultrasound studies confirms the effectiveness of hydroxyapatite in the decontamination of zinc-polluted water.

The present research is part of the recent concern about the use of hydroxyapatite as an adsorbent for metals in water. Due to its high affinity for divalent heavy metals ions, hydroxyapatite is a unique inorganic compound and has been used to remove various heavy metals such as nickel [56] lead [56,57,58], cadmium [59], cooper [60], zinc [8,61] and arsenic [62]. In their study, Ramesh et al. [4] reported that HAp had a greater affinity towards cooper ions compared to zinc ions, estimating a removal capacity of 125 mg of Cu/g and 30.3 mg of Zn/g. Meanwhile, Chen et al. [62] highlighted that the sorption affinity of HAp for lead ions in single-metal sorption systems was significantly higher than for copper and cadmium ions. Regarding the adsorption capacity of zinc ions onto hydroxyapatite, studies have reported values in the range of 19.76–109.37 mg/g [63,64]. The studies presented above are in good agreement with the results obtained in this research regarding the use of hydroxyapatite powders in the removal of zinc ions from contaminated aqueous solutions.

In conclusion, we could say that the hydroxyapatite has proven to be a good absorbent for zinc ions and could also be used for other pollutants. Furthermore, hydroxyapatite has additional benefits such as low costs and being easy to obtain.

## 4. Conclusions

This study investigated the efficiency of hydroxyapatite obtained by an adapted coprecipitation method in the removal of zinc from aqueous solutions. During the process of removing zinc ions from aqueous solutions, zinc ions were introduced into the HAp lattice. After introducing zinc ions into the HAp lattice, a slight change in crystallinity and a decrease in particle size was observed. The adsorption of zinc ions from aqueous solutions was evidenced by XRD, SEM, and TEM. Hydroxyapatite has good affinity for Zn. The effectiveness of hydroxyapatite in the decontamination of zinc-polluted water was confirmed for the first time by ultrasound studies. The decontaminated water has ultrasonic characteristics such as velocity and signal aspect closely resembling those of pure water. The sorption of Zn onto HAp was well characterized by the Langmuir and Freundlich models. The results revealed that the maximum adsorption capacity for the solid phase calculated from the linearized form of Langmuir equation, q_m_, for Zn^2+^ was 57.504 mg/g and the Langmuir constant K_L_ for the adsorption of Zn^2+^ was 0.052 L/mg. Furthermore, for the first time, the effectiveness of hydroxyapatite in the adsorption of zinc ions from contaminated solutions was highlighted by ultrasonic measurements. The results obtained from ultrasonic measurements were also in good agreement with those obtained from adsorption experiments.

The results obtained in the present study show that HAp nanopowders are good adsorbents for zinc ions and can therefore be considered for applications for removal of zinc ions from wastewater.

## Figures and Tables

**Table 1:** The percentage of zinc ions removal and adsorption capacity of HAp upon varying the zinc concentrations.

Zinc Concentration (mg/L)	% Removal of Zinc Ions	Adsorption Capacity q_e_ (mg/g)
10	34.65	3.47
20	35.4	7.08
30	39.97	11.99
50	40.36	20.18
70	40.77	28.54

**Table 2:** Isotherms parameters for zinc adsorption onto HAp.

Pollutant	Sample	Langmuir			Freundlich		
		q_m_ (mg/g)	K_L_ (L/mg)	R^2^	N	K_F_	R^2^
Zn^2+^	HAp	57.504	0.052	0.995	0.884	0.408	0.997
